# Animal Models of Human Disease

**DOI:** 10.3390/ijms242115821

**Published:** 2023-10-31

**Authors:** Sigrun Lange, Jameel M. Inal

**Affiliations:** 1 Tissue Architecture and Regeneration Research Group, Department of Biomedical Sciences, University of Westminster, London W1W 6UW, UK; 2 Pathobiology and Extracellular Vesicles Research Group, Department of Biomedical Sciences, University of Westminster, London W1W 6UW, UK; 3 Cell Communication in Disease Pathology, School of Human Sciences, London Metropolitan University, London N7 8DB, UK; j.inal@londonmet.ac.uk; 4 Biosciences Research Group, School of Life and Medical Sciences, University of Hertfordshire, Hatfield AL10 9EU, UK

**Keywords:** animal models, pathobiology, chronic disease, regeneration, infectious disease, cancer, autoimmunity, neurodegenerative disease, liquid biopsy, biomarkers

## Abstract

The use of animal models of human disease is critical for furthering our understanding of disease mechanisms, for the discovery of novel targets for treatment, and for translational research. This Special Topic entitled “Animal Models of Human Disease” aimed to collect state-of-the-art primary research studies and review articles from international experts and leading groups using animal models to study human diseases. Submissions were welcomed on a wide range of animal models and pathologies, including infectious disease, acute injury, regeneration, cancer, autoimmunity, degenerative and chronic disease. Seven participating MDPI journals supported the Special Topic, namely: *Biomedicines*, *Cells*, *Current Issues in Molecular Biology*, *Diagnostics*, *Genes*, the *International Journal of Molecular Sciences*, and the *International Journal of Translational Medicine*. In total, 46 papers were published in this Special Topic, with 37 full length original research papers, 2 research communications and 7 reviews. These contributions cover a wide range of clinically relevant, translatable, and comparative animal models, as well as furthering understanding of fundamental sciences, covering topics on physiological processes, on degenerative, inflammatory, infectious, autoimmune, neurological, metabolic, heamatological, hormonal and mitochondrial disorders, developmental processes and diseases, cardiology, cancer, trauma, stress, and ageing.

The use of animal models of human disease is critical for furthering our understanding of disease mechanisms, for the discovery of novel targets for treatment, and for translational research [1,2,3]. While cellular models are commonly used in the first instance for elucidating mechanistic pathways in disease processes, the use of animal models to study human pathologies and clinical features is invaluable for pre-clinical research [4,5]. As animal research is under increasing scrutiny, the scientific community must critically evaluate ethical use of the most appropriate animal models for the given pathology, including for pre-clinical investigations and testing of new treatments [6,7]. Common laboratory models for human disease encompass species lower in the phylogeny tree, including the nematode *Caenorhabditis elegans* [8,9,10,11], the fly *Drosophila elegans* [12,13,14,15], zebrafish (*Danio rerio*) [16,17,18,19], and the amphibian *Xenopus laevis* [20,21,22,23]. Murine models, particularly mice and rats, are standard models used for a wide range of disease modelling [24,25,26,27,28], whereas larger animal models, such as dogs, pig, sheep, and non-human primates, which more closely resemble human anatomy and physiology are desirable for translational studies in cardiovascular, metabolic, genetic (including rare disease), and neurodegenerative diseases [29,30,31,32,33,34,35].

The Special Topic entitled “Animal Models of Human Disease” (https://www.mdpi.com/topics/Model_Animals (accessed on 15 October 2023)) was launched to create an overview of currently used animal models, by publishing state-of-the-art primary research studies and review articles from international experts and leading groups using animal models to study human diseases. In total, 44 papers were published in this Special Topic, with 36 full length original research papers, 2 research communications and 6 reviews. The following original research papers were published in this Special Topic and are briefly summarized below:

The study by Colvin and coworkers examined baseline lung and heart biology of the Dp16 mouse model of Down syndrome, identifying baseline gene expression in lung and hearts for key parameters related to lung infection susceptibility (Contribution 1). Assessment of pulmonary and cardiac function reveled increased lung stiffness with bronchus associated lymphoid tissue; changes in mRNA of triplicated genes in lung; hypotonicity of heart ventricles; unique blood plasma cytokine and chemokine signatures; and sex-specific changes in interferon and STAT3. These findings highlight the translatable use of the DP16 mouse model for recurrent severe respiratory tract infection in human Down syndrome.

Bullock and coworkers reported a canine case study with progressive neurological disease and global brain atrophy, identified to have a homozygous *MAN2B1* missense mutation (Contribution 2). Findings identified accumulations of vacuolar inclusions in brain cells as well as secondary storage bodies due to α-mannosidase deficiency, resulting in impairment of the autophagolysosomal system. Signs of glial activation indicative of neuroinflammation in brain and spinal cord were also reported. This canine case highlights similarity between species in lysosomal storage disease and may further understanding to develop therapeutic treatment approaches.

In Contribution 3, Ariozono and collaborators assessed the potential of oral nicotinamide riboside treatment for axonal protection in glaucoma degeneration. The study used a rat model of ocular hypertension and demonstrated axonal protection by morphometric analysis of optic nerve cross sections and showed that retinal ganglion cell fiber loss was prevented in response to nicotinamide riboside administration. A role for p-AMK upregulation is highlighted as a possible mechanism in NR-mediated protection against glaucomatous RGC axonal degeneration.

Cueto-Ureña and co-authors highlighted sex-dependent patterns associated with glutathione peroxidase gene expression in brain tumours (Contribution 4). The authors used an experimentally transplacental-induced rat brain tumour model, assessing possible sex differences relating to tumour tissue. Findings highlighted tumour associated changes in gpx gene expression, which showed sex-specific differences. This study furthers understanding of molecular mechanisms in brain tumours and the biomarker potential of glutathione peroxidase gene expression patterns in the clinic.

In Contribution 5, Chi-Cheng and team assessed stress-induced immunosuppression in mouse models of physiological regulations during psychological stress. A restraint stress model as well as cold exposure model were utilized. Stress hormones were found to be increased in the restraint stress animals and these also showed different immunosuppression from the cold exposure model as observed by increased splenic macrophage cell death. Inflammasome inhibitors ameliorated restraint–stress-induced spleen infiltration and macrophage cell death. The findings highlight the need for in-depth investigations into molecular mechanisms underlying different stressors and associated responses and may have implications for targeted treatments for stress-induced immunosuppression.

In Contribution 6, Wo and coworkers identified that histone deacetylase inhibitor suppresses glycolysis and can reduce mortality in sepsis. The study utilized an LPS-induced mouse model of endotoxemia to mimic early stages of systemic inflammation and the histone deacetylase inhibitor suberoylanilide hydroxamic acid. Roles for acetylation of lactate dehydrogenase and associated reduction in glycolysis were identified. This was accompanied by reduced inflammasome activation observed in bone-marrow-derived macrophages in response to SAHA administration. This study lays foundations for targeting lactate dehydrogenase in sepsis treatment.

The study by Riedl and colleagues provides a characterization of a mild atopic dermatitis mouse model, induced by 2,4-dinitrochlorbenzene (DNCB) (Contribution 7). Challenges and reproducibility of appropriate murine models and translatability to human are discussed. This study establishes and describes a detailed procedure for the generation of a DNCB-induced mouse model, showing it to display many features observed in mild human-like extrinsic atopic dermatitis, including a comprehensive spectrum of parameters which shows a local inflammatory skin milieu. The study adds a new research tool and furthers current understanding of mechanisms and treatment strategies for human atopic dermatitis.

In Contribution 8, Rahavi and collaborators generated an adapted mouse model for high-risk non-metastatic neuroblastoma, using a novel luciferase-expressing derivative of the Th-MYCN mouse. A transplantation-based approach allowed for longitudinal monitoring of luciferase-tagged, transplanted primary tumour cells from disease-bearing mice to immune-deficient or immune-competent animals. This approach overcomes previous limitations of this model for the evaluation of NB growth and regression at secondary sites. The approach can provide a useful platform for the evaluation of treatment responses and optimization of immune-based treatments.

Rousseau and colleagues explored seizure related variants in the ATP6V1B2 mouse model (Contribution 9). They generated and assessed the Atp6v1b2emR506* heterozygous mouse model and showed that is a valuable tool to explore both the pathophysiology and potential treatments for vacuolar ATPases-associated epilepsy and neurodevelopmental disorders. The response to pentylenetetrazol treatment was also assessed in both heterozygous (similar to human patients) and homozygous mice. This study provides a valuable tool to further explore pathophysiology and potential treatments for vacuolar ATPases-associated epilepsy and disorders.

Mitra and coworkers provided the first study exploring the biomarker potential of extracellular vesicles (EVs) in neonatal seizures (Contribution 10). The team assessed extracellular vesicle (EV) signatures, including citrullinated protein cargoes, in a piglet model of neonatal seizures, also assessing response to phenobarbitone treatment. EV signatures were assessed in both plasma and cerebrospinal fluid, with more marked changes observed in plasma-EV signatures. Findings indicate peripheral responses to the insult at the early time point assessed in this study. The study highlights the clinical biomarker potential EVs in neonatal seizures, where effective treatment and reliable biomarkers are still lacking.

In Contribution 11, Patil and team used system biology approaches to identify a suitable chemically induced liver cirrhosis model that could parallel Hepatitis B Virus pathophysiology (Contribution 11). The study revealed that a considerable number of genes modulated by HBV are common with alcohol and lipopolysaccharide-induced hepatitis. Based on the reported findings, the authors propose that alcohol-induced hepatitis can be a suitable rodent model to study chronic HBV infection and that LPS-induced hepatitis may be used to model acute inflammatory responses to HBV.

In Contribution 12, Vallés-Saiz and collaborators assessed protective effects of lamivudine (3TC), a reverse transcriptase inhibitor, on reducing taupathy in and FTDP-17-tau overexpression mouse model of Alzheimer’s disease. Findings showed that lamivudine treatment attenuated motor deficits and improved short-term memory, alongside decreased histopathological changes associated with taupathies. The study is therefore of importance for new treatment options, with the possibility of repurposing lamivudine, which is used to treat HIV, for potential therapeutic application in early symptoms of neuropathology.

Contribution 13 is a study by Shah and collaborators, who generated a novel mouse CRISPR/Cas9 knockout model, which partially captures the human phenotype of Fanconi anaemia (FA). In addition, cells derived from the model, as well as their reconstituted counterparts, provide an opportunity to study the FA pathway, particularly the role of FANCG under specific ICL stress conditions caused by metabolites as well as chemotherapeutics. This study is therefore of considerable value for both in vivo and in vitro studies of FA.

The study by Siwakoti and team identified roles for activating transcription factor 3 (ATF3) in metformin mediated protection in psychological-stress-associated gastrointestinal tract injury (Contribution 14). The study used a genetically deficient ATF3 KO mouse model, to show GI protective effects and metformin-mediated rescue in an ATF3-dependent manner. The study highlights roles for ATF3 as a regulator for metformin-mediated rescue of stress-induced GI tract injury, elucidating novel molecular mechanisms in this pathology.

In Contribution 15, Haque and collaborators showed that deficiency of adipose aryl hydrocarbon receptor (AhR) protects against diet-induced metabolic dysfunction through sexually dimorphic mechanisms. The team used a mouse model with conditional AhR depletion, showing that CadKO facilitates a lean phenotype in females and mediates healthy adipose–hypothalamic crosstalk. In males, adipose-specific AhR depletion delays the development of obesity and insulin resistance through the maintenance of healthy crosstalk between adipocytes and immune cells. The study provides valuable insights into mechanisms relating to sex-dependent differences in metabolic diseases.

Lin and team carried out a comparative study in three different species of mice to assess skin changes in chronic photoaging models (Contribution 16). The results showed differences in response to chronic photoageing, based on histological assessment. This study highlights appropriate murine models for the assessment of different aspects in exogenous skin ageing, providing a reference for the establishment and selection of animal models for chronic skin photoaging studies.

In Contribution 17, Lien and coworkers investigated the role for the Dengue virion-associated envelope protein domain III (EIII) in hemorrhage pathogenesis, using murine models and in vitro assays. EIII-coated silica nanoparticles (EIII-SNPs) were compared to EIII or silica nanoparticles alone for cytotoxic effects on endothelial cells and hemorrhage pathogenesis in mice. This study highlights the potential role of EIII in dengue hemorrhagic fever pathogenesis and the presented model may pave way for further progress in therapeutic strategies for severe dengue infections.

Liu and team performed morphologic and genetic analyses of mammary tumors arising from MMTV-PyVT mice, a mouse strain in which the oncogenic polyoma virus middle T antigen is driven by the mouse mammary tumour virus promoter (Contribution 18). Histological and whole mount carmine alum staining analysis was carried out on tumours at various stages and whole-exome sequencing was used to identify constitutional and tumor-specific mutations, identifying possible pathogenic frameshift indels of *Muc4*. The findings of the study validate MMTV-PyVT transgenic mice as a multistage model for mammary carcinoma development and progression.

In Contribution 19, Gusev and collaborators investigated the reorganization and suppression of store-operated calcium entry in podocytes in type 2 diabetes (DM2), utilising a rat in vivo model and human podocyte cells. Findings of the study provide new insights into the mechanism of store operated Ca^2+^ entry organization in healthy podocytes and after DM2 development. This is of relevance for the development of pharmacological treatment in early stages of diabetic nephropathy.

Dong and collaborators optimized a monobenzone-induced vitiligo mouse model by the addition of chronic stress modelling (Contribution 20). The pathogenesis of vitiligo, a skin depigmentation disorder, is complex and unclear. The team addressed challenges in current vitiligo models, and added mental factors, using a model of chronic unpredictable stress. Findings showed that added chronic unpredictable mild stress stimulus increased skin depigmentation and altered metabolic pathways. This combinatory stress and vitiligo model will aid study and evaluation of vitiligo drugs.

In Contribution 21, Watanabe and team generated an animal model of isoleucyl-tRNA synthetase 1 (IARS1) mutation-related disorder, which is relevant for pediatric mitochondrial disease. The mouse model replicated relevant pathophysiology, including growth retardation and hepatic steatosis, observed in preweaning humans and cattle. The mutant mice suffered from mitochondrial hepatopathy, and similar findings were confirmed in human HepG2 cells with siRNA knockdown of the IARS1. Proteomic analysis identified proteins associated with mitochondrial hepatopathy and lipid metabolism. This study has generated a new mutant mouse model for advancing research of IARS disorder.

Chen and colleagues, developed an evaluation model for osteoarthritis treatment, assessing typical markers to determine the relationship between cartilage histology, epiphyseal trabecular bone, and age. In Contribution 22, the team further assessed GRGDS peptides as a potential treatment for osteoarthritis, using a mouse model of spontaneous osteoarthritis. Animals of different age groups, with and without GRGDS treatment, were compared, identifying a range of age-related changes that can partly be mitigated by GRGDS treatment. Findings of the study present evaluation methods to characterize and measure the efficacy of cartilage damage treatments in the STR/ort mice with spontaneous OA.

In Contribution 23, Vincent and collaborators carried out retinal phenotyping of Lafor disease, a progressive neurological disorder The team utilized an Epm2a^−/−^ knockout mouse model, assessing the animals at 10 and 14 months in vivo by electroretinogram testing, optical coherence tomography and retinal photography. The team furthermore performed ex vivo retinal assessment to quantify Lafora body deposition in the eye only. The degree of retinal involvement in this mouse model was found to be milder than in the patients, but the model is a useful tool that lays foundations for future studies, including assessing treatment options for Lafora body deposition in the retina ex vivo. The model can also be useful for in vivo monitoring of neurological and behavioural deficits.

Kalinina and co-workers assessed physiological and functional effects of dominant active TCRα expression in transgenic mice (Contribution 24). The team analyzed the functional status of the immune system, with findings suggesting that TCRα transgene expression may delay thymic involution and maintain TCRβ repertoire diversity in old transgenic mice. Changes detected in the systemic homeostasis in 1D1a transgenic mice were minor and primarily transient and may indicate variations in the ontogeny of wild-type and transgenic mouse lines.

In Contribution 25, Zhang and collaborators used a murine model for rosacea, a chronic inflammatory skin disease with limited reports in animal models to date, particularly regarding late manifestations. The team monitored skin lesions and major histopathological changes in a rosacea-like phenotype in Balb/c mice, induced by prolonged LL-37 administration. The study shows that irreversible rosacea-like lesions can be induced by long-term LL-37 administration and provides a tool for establishing a model of rosacea fibrosis and advanced lesions in the treatment of rosacea.

Li and collaborators assessed transcriptomic profiles within M1 and M2 phenotypes of macrophages in a pig model (Contribution 26). Findings indicated that porcine M1 and M2 macrophages have gene signatures consistent with human and mouse macrophage phenotypes, respectively. GSEA analysis revealed four types of macrophages with different functions and highlights the potential of macrophage signatures to discriminate between different pathogenic infections. This study provides a new model for predicting immune responses.

In Contribution 27, Hu and collaborators assessed roles of RhoA signalling in pediatric end-stage renal failure caused by nephronophthisis (NPHP), using mouse knockout models. Findings showed alleviated renal pathological changes in the mouse model. EMT changes in NPHP1KD HK2 cells were reversed by GEF-H1 knockdown in vitro. The results indicate that the GEF-H1/RhoA/MLC2 axis plays a key role in the pathogenesis of NPHP1-defective NPHP, laying the foundation for further in-depth research.

Tassinari and collaborators reported the development of rodent models that mimic gender-affirming hormone therapies and report novel biomarkers of sex transition (Contribution 28). The team aimed to set appropriate hormone therapy doses to implement transgender rat animal models and to identify specific associated biomarkers. Demasculinizing-feminizing models were generated using β-estradiol plus cyproterone acetate, while defeminizing-masculinizing models were exposed to testosterone. Sex-specific CYP gene expression was identified as a biomarker supporting the set of proper (de)masculinizing or (de)feminizing hormone therapy to obtain a reliable animal model for transgender people. This study is of considerable value for research relating to hormone therapies and risk assessment for transgender people.

In Contribution 29, Borchardt and colleagues used *Drosophila melanogaster* to study the effects of ether and non-ether volatile anesthetics on the mitochondrial electron transport chain. The goal of the study was to identify whether the alkane volatile anesthetic halothane and other mutations in Complex I and in Complexes II–V of the mitochondrial electron transport chain cause anesthetic-induced neurotoxicity. Findings showed that both fly lines were susceptible to toxicity from halothane, that alleles of accessory subunits of Complex I predisposed to anesthetic-induced neurotoxicity and that mutations in Complexes II–V did not result in an anesthetic-induced neurotoxic phenotype. Furthermore, in wild-type flies, halothane was toxic under anoxic conditions. The authors concluded that anesthetic-induced neurotoxicity is neither limited to ether anesthetics nor exclusive to mutations in core subunits of Complex I.

Zhang and coworkers assessed CDCR3-mediated immune regulation and inflammatory responses in authophagy during intestinal injury (Contribution 30). The team used an LPS-induced inflammatory CXCR3 knockout mouse model, showing reduced structural damage in the intestinal mucosa, and increased right junction protein expression. The CXCR3 knockout also reduced LPS-induced increase in inflammatory factors and enhanced autophagy markers in vivo. Mechanistic studies were further carried out in cellular models. The findings provide information on immunoregulatory mechanisms in intestinal dysfunction and highlight investigations into therapeutic approaches targeting CXCR3.

In Contribution 31, Gerstner and coworkers investigated roles for Arf-like Protein 2 (ARL2), a multifunctional protein across phylogeny, in the control of microtubule neogenesis during early postnatal photoreceptor development. The group generated a mouse model with conditionally deleted ARL2 in the retina during embryonic development or at later stages after ciliogenesis. Findings of the study suggest that ARL2 and dynein depend on each other to generate a functional microtubule cytoskeleton during early photoreceptor development. The study provides important information for molecular mechanisms in photoreceptor development.

The study by Wu and coworkers evaluated the effects of ketamine inhalation on behavioural and lower urinary tract changes in a mouse model, mimicking human ketamine abusers (Contribution 32). The inhalation model was also compared to intraperitoneal ketamine administration models. Findings showed that ketamine inhalation induces behavioral and lower urinary tract changes that are comparable to intraperitoneal ketamine injections. The study is important for human ketamine abuse research and importantly the inhalation model provides a more animal-friendly approach for modelling ketamine abuse.

In Contribution 33, Hu and collaborators aimed at identifying factors involved in the unusual control of high blood glucose levels in chicken and to assess putative protective effects on Type 2 Diabetes Mellitus (T2DM) in a rat model. Overall, the study demonstrated that the serum metabolite of DL-arginine can maintain blood glucose homeostasis and suppress inflammatory response in chickens. The study therefore highlights a novel target for developing therapeutic agents to regulate hyperglycemia.

Cristancho and coworkers studied sex-specific and age-specific differences in anxiety and learning in children exposed to prenatal inflammation, with a focus on possible behavioural changes due to in utero inflammation (Contribution 34). Using a mouse model of LPS-induced in utero in an inflammation mouse model, assessment was carried out on neonatal, juvenile, and adult mice. Findings showed that the prenatal inflammatory exposure caused differences in sex-dependent responses at different stages of development, including in gene expression patterns for hippocampal neurogenesis. The study brings important understanding on intrauterine inflammation and its downstream effects on age- and sex-specific effects on altered behaviours, and paves the way for novel therapeutic interventions to improve long-term outcomes.

In Contribution 35, Cassano and coworkers examined the link between metabolic disturbances and cognitive decline, using a Type 2 Diabetes Mellitus (T2DM) rat model. The antianginal drug ranolazine, which also acts as a neuroprotective drug, was used, also in combinatory treatment with the diabetes drug metformin. Hippocampal neurodegeneration assessment showed improved T2DM-induced neuronal loss and neuronal damage in both ranolazine and metformin treated animals. Astrocyte activation also showed lower signals in the treated diabetic animals. The study highlights the potential of using ranolazine for treatment in T2DM-associated cognitive decline.

The study by Zhao and collaborators presented a new strategy for producing stem cell complementation-based organ regeneration (SCOG) host embryos that may be a useful tool to study lung regeneration (Contribution 36). The team developed a novel lung vacancy mouse model by deleting thyroid transcription factor 1 (TTF-1) Exon 2, involved in embryonic development and differentiation of the thyroid and the lungs. To overcome challenges of previous mouse models, the team used CRISPR/Cas9 to produce TTF-1 knockout (E2del) mice. 57% of the E2del embryos presented desirable features including type I tracheal agenesis (TA) and bilateral sac-like lungs. This new model will be useful for application in lung regeneration studies.

In Contribution 37, Yuan and team profiled tRNA-derived small RNAs (tsRNAs) in a mouse model of septic cardiomyopathy. The study identified 101 up-regulated and 57 down-regulated tsRNA targets, overall showing that tsRNAs can protect cardiomyocytes during the development of septic cardiomyopathy and reduce cardiomyocyte death. The study lays foundations for further in-depth research on the identified tsRNA candidates linked to septic cardiomyopathy and therapeutic potential in myocardial injury.

The study by Guo and collaborators analysed the potential of developing pig models for β-thalassemia (Contribution 38). The team reported a hemoglobin-encoding gene, in pigs, which showed high amino acid conservation to humans. The evolutionary relationship of hemoglobin-encoding genes was compared in humans, pigs, and mice, showing that the β-chain structure of pigs and humans was highly similar. The hemoglobin-encoding gene expressions showed significant differences in the spatiotemporal expression patterns in three developmental stages of six different pig breeds. The study provides an important theoretical basis for further construction of a gene-edited pig model to study β-thalassemia and putative treatments.

In Contribution 39, Duperray and team used the amphibian vertebrate model *Xenopus laevis* to assess developmental requirements for purine metabolism. The team provides the first functional characterization of purine pathway genes, and generated morphants, showing expression and function in nervous and muscular embryonic tissues. Findings of the study identified critical functions of the purine biosynthetic pathway genes in myogenesis during vertebrate embryogenesis. The model generated in the study lays foundations for further functional studies identifying molecular mechanisms in neuromuscular tissue development and may be translatable to patients with purine deficiency.

In addition to original research papers, seven reviews were published in this Special Topic:

Briguglio and co-authors contributed a comprehensive review on neurodevelopmental and neurodegenerative disease related roles of Ataxia–Telangiectasia Mutated (ATM), a serine/threonine protein kinase principally known to orchestrate DNA repair processes (Contribution 40). Mutations in the ATM gene lead to a recessive disorder, Ataxia-Telangiectasia (AT), which is characterized by ataxic movements due to cerebellar atrophy or dysfunction. This is accompanied by immune alterations, genomic instability and furthermore predisposition to cancer. AT phenotypes range from neurological abnormalities and cognitive impairments to neuropsychiatric features. This review focusses on the roles of ATM in neuronal physiology and pathology, also discussing structural and functional changes in hippocampal and cortical synapses of AT mouse models. The paper is important for state-of-the-art knowledge of ATM-dependent mechanisms in pathological mechanisms in neurodevelopmental and degenerative disorders.

The review on marmoset cell lines by Bayurova and co-authors (Contribution 41) discusses their availability and applications in biomedical research. Common marmosets (*Callithrix jacchus*) are widely used in biomedical research as a small non-human primate model. Before moving into in vivo models, early research is often carried out in cell cultures derived from different marmoset tissues, important for the 3Rs. The authors discuss current challenges of standardization and validation in marmoset cell lines and provide a comprehensive summary on advantages, drawbacks and research applications of the marmoset cell lines published to date. This review paper is important for pushing the development of new standardized and commercially available marmoset cell lines, with increased translational potential and reproducibility.

In Contribution 42, Tseng and co-authors reviewed animal models for hearth transplantation, covering multifaceted challenges on topics of immunopathology of graft rejection, immunosuppressive therapies, anastomotic techniques, and graft preservation techniques (Contribution 42). The authors discuss advantages of small experimental animal models, which also can be genetically modified, while challenges of these models relate to lack of clinical translatability. Discussion on larger animal models includes canines, pigs, and non-human primates, which are often used to validate results obtained from small animal studies due to advantages relating both to anatomy and physiology, and their use to assess feasibility of experimental therapies to clinical practice. The paper is a valuable up-to-date resource on current animal models for heart transplantation, including with a focus on pathological conditions relating to each model.

The review by Basheer and co-authors discusses the suitability of zebrafish as a relevant genetic model for human primary immunodeficiency disorders (PIDs) (Contribution 43). The conservation of the zebrafish immune system is explained and examples of zebrafish models for various human PIDs are provided, including for autoinflammatory disorders, multi-system immunodeficiency, severe combined immunodeficiency, combined immunodeficiency, neutropenia and defects in leucocyte mobility and respiratory burst. The review furthermore discusses diverse applications of the zebrafish models in immunology, microbiology, regenerative biology, and oncology. The review is a valuable contribution to understanding the strengths and current limitations in zebrafish modelling for human PIDs.

In Contribution 44, Blangy-Letheule and co-authors reviewed spontaneous sepsis from the perspectives of veterinary to human medicine. The authors discuss current challenges of reliable predictive biomarkers and therapeutic targets in sepsis and the need for appropriate experimental animal models. The limitations of mouse models regarding translatability to humans is discussed, and the authors highlight the potential of using horse models as a large animal model for spontaneous sepsis, with more appropriate translatability to humans. The review provides a comprehensive overview of different animal species used to model human sepsis, with an in-depth focus on adult equine sepsis and highlights its potential in veterinary and human medicine.

The review by Nikam and co-authors summarises state-of the art knowledge on animal models of abusive head trauma and associated biomarkers (Contribution 45). The authors highlight the challenges of developing experimental animal models that can simulate clinical cases of abuse head trauma. Animal models which have been designed to mimic behavioral and pathophysiological pediatric abusive head trauma are critically discussed, including rodents, piglets, lambs, and non-human primates. Challenges in clinical translatability of the different animal models to human infants are furthermore highlighted in relation to secondary injuries, long-term effects, and degenerative disease. The review provides a comprehensive overview of the use and value of animal models for different head trauma and the identification of pre-clinical biomarkers.

In Contribution 46, Patiño-Morales and co-authors reviewed the use of the chicken embryo chorioallantoic membrane assay for modelling neuroblastoma biology. Technical challenges are critically discussed, and main findings summarised for research application of the model to study angiogenesis, metastasis, drug sensitivity, and drug resistance. The authors furthermore highlight the importance of the assay for the 3R principles and its clinical usefulness as an alternative animal model in advancing understanding of tumour biology.

The publications which contribute to this Special Topic provide a wide range of original research studies and reviews, which cover clinically relevant, translatable, veterinary, and comparative animal models. These papers emphasize the importance of using diverse animal models, as well as animal-derived cell lines, including from larger animal models, to inform human disease and further current understanding of fundamental pathways in a wide range of pathologies. The reported findings in this Special Topic, continue paving way to aid in discovery of new therapeutic targets in inflammatory, infectious, autoimmune, neurological, metabolic, heamatological, and mitochondrial disorders, in developmental processes and diseases, cardiology, cancer, trauma, ageing, and stress-induced pathologies. The common use of murine models in biomedical research is reflected in the contribution of 33 original research articles in this Topic, using mouse or rat models, whereas three studies used pigs, one used dogs, one used flies, two papers focused on chicken models, and one on zebrafish. The use of non-human primates both in vivo and in vitro was also discussed. Given the importance of adhering to the 3Rs (Replacement, Reduction, and Refinement) in animal research, future strategies in pre-clinical research will need to further focus on the development of sophisticated cellular tools, including organoids, organ-on-chip tools, microfluidics, and standardized cell lines from a wider range of animal model species [36,37].
